# Should Contralateral Nodules Be an Indication of Total or Completion Thyroidectomy for Patients With Unilateral Papillary Thyroid Carcinoma?

**DOI:** 10.3389/fendo.2021.723631

**Published:** 2021-08-09

**Authors:** Tengfei Ma, Haiyang Wang, Jifeng Liu, Jian Zou, Shixi Liu

**Affiliations:** Department of Otolaryngology Head & Neck Surgery, West China Hospital, Sichuan University, Chengdu, China

**Keywords:** papillary thyroid carcinoma, contralateral nodules, lobectomy, total thyroidectomy, completion thyroidectomy, recurrence, clinical contralateral PTC

## Abstract

**Objective:**

To determine whether papillary thyroid carcinoma (PTC) patients with benign or nonsuspicious nodules in the contralateral lobe have a higher rate of recurrence or worse survival after lobectomy compared to those without nodules in the contralateral lobe.

**Methods:**

Adult patients who underwent lobectomy and were diagnosed with unilateral PTC (2013-2015), were identified from an institutional database. Patients who previously had cytologically benign nodules or nonsuspicious nodules in the contralateral lobe comprised the contralateral nodule (CN) group. Patients who did not have nodules in the contralateral lobe comprised the unilateral nodule (UN) group.

**Results:**

370 patients were included: 242 in the UN group and 128 in the CN group. After a median follow-up of 62 months (range, 16–85 months), recurrence was confirmed in 4.1% patients in the UN group and 5.5% patients in the CN group (*p* = 0.559). Clinical contralateral lobe PTC was detected in 2.9% (7/242) of patients from the UN group and 3.9% (5/128) of patients from the CN group (*p* = 0.601). The 5-year contralateral lobe recurrence-free survival (RFS) rates were 96.8% in the UN group and 97.4% in the CN group (*p* = 0.396). The 5-year loco-regional RFS rates were 98.4% in the UN group and 97.8% in the CN group (*p* = 0.690). The 5-year disease-specific survival rates were both 100%.

**Conclusion:**

PTC patients with benign or nonsuspicious CNs have similar recurrence and survival rates after lobectomy compared to those without CNs. CNs alone should not be an indication for total or completion thyroidectomy.

## Introduction

The prevalence of thyroid nodules among asymptomatic persons is about 19–70% (1–3). About 8–16% of thyroid nodules are malignant (1), of which papillary thyroid cancers (PTCs) are the most common (4, 5). In the past few decades, total thyroidectomy has been the main treatment for PTC. However, in recent years, many experts have called for less aggressive treatment of PTC, as studies have shown that total thyroidectomy does not provide a better clinical outcome than unilateral lobectomy for patients with low or intermediate risk PTC (6–9). In agreement with this conservative trend, most clinical guidelines currently recommend lobectomy as a valid treatment option for patients with a tumor < 4 cm, without gross extrathyroidal extension (ETE), and non-metastatic disease (10–12).

The incidence of thyroid cancer, mainly PTC, has increased sharply worldwide over the past 30 to 40 years (13, 14). Given the striking prevalence of thyroid nodules and the increasing incidence of PTC, it is common to detect bilateral thyroid nodules by ultrasonography (US) in patients with pathologically confirmed PTC in one lobe (15).

For patients diagnosed with unilateral PTC, nodules in the contralateral thyroid lobe have been considered a factor influencing the extent of surgery. Indeed, according to the 2015 American Thyroid Association (ATA) guidelines and the National Comprehensive Cancer Network guidelines (version 2.2020), the presence of contralateral thyroid nodules in PTC patients may be criteria for recommending total or completion thyroidectomy to better address suspicions of bilateral disease and facilitate follow-up (11, 12). Several studies may favor these recommendations, since up to 48% of patients diagnosed with unilateral PTC have PTCs in the contralateral lobe (16, 17). However, these studies have focused on patients who underwent total thyroidectomy or completion thyroidectomy. The natural follow-up outcomes of patients with unilateral PTC and contralateral nodules (CNs) after lobectomy have rarely been studied. A recent study by Ritter et al. (15) showed that in PTC patients with benign or nonsuspicious CNs under close follow-up, the rate of contralateral lobe PTC after lobectomy was only 5%.

Accordingly, we hypothesize that lobectomy is an appropriate treatment for low-risk PTC patients (tumor <4 cm, non-gross ETE, and non-metastatic disease) with contralateral thyroid lobe nodules. Therefore, we determined whether PTC patients with nonsuspicious CNs have worse outcomes after lobectomy.

## Materials and Methods

### Study Cohort

This study included adult patients (≥18 years), who underwent lobectomy and were diagnosed with unilateral PTC by postoperative pathology from January 2013 to December 2015 at West China Hospital of Sichuan University. The extent of surgery was at the discretion of the treating surgeon with consideration for patient preference. In our hospital, ipsilateral prophylactic central-compartment neck dissection was performed for all patients with PTC. Exclusion criteria were: primary tumor size ≥ 4 cm, gross ETE, aggressive histology (tall cell, columnar cell, and hobnail variants) and/or vascular invasion, clinical cervical lymph node metastasis, and evidence of distant metastasis. Included patients had at least 12 months follow-up after surgery. Patients were categorized into two groups. Patients who did not have nodules in the contralateral lobe comprised the unilateral nodule (UN) group, and patients who previously had cytologically benign nodules defined by fine needle aspiration or nonsuspicious nodules by US in contralateral lobe comprised the CN group. The risk of malignancy of the contralateral thyroid nodules was estimated according to the sonographic pattern classification proposed by the 2015 ATA guidelines (11). Nonsuspicious nodules were defined as nodules without ATA high-risk sonographic features (microcalcifications, irregular margins, taller than wide shape, rim calcifications with small extrusive soft tissue component, and evidence of ETE). All patients were followed with thyroid and neck US every 3 months in the first year after initial surgery; if disease-free, they were followed up every 6 months in the second year and then annually since the third year. Disease-free was defined as the absence of suspicious findings on the neck US. During the follow-up period, fine needle aspiration (FNA) was generally recommended for patients with suspicious findings on neck US. Referring to previous literature, recurrence after unilateral thyroid lobectomy was classified as recurrence in the contralateral lobe and loco-regional (ipsilateral thyroid bed or cervical lymph nodes) recurrence (18). Patients with confirmed or suspicious contralateral lobe recurrence were recommended to receive completion thyroidectomy and central-compartment neck dissection. Selective neck dissection was performed for those with confirmed or suspicious loco-regional recurrence.

The following data were retrospectively reviewed and analyzed: age at diagnosis, gender, tumor size, microscopic ETE condition, and tumor stage. Tumor stage was classified according to the American Joint Committee on Cancer (AJCC) 8th Edition TNM staging system (19). The data of postoperative complications including voice impairment (hoarseness, voice fatigue, low-pitch voice), dysphagia, and hypocalcemia was recorded. The information of voice impairment and dysphagia were obtained through physicians’ active inquiry. Hypocalcemia was defined as serum calcium below the lower limit of the normal range at our institution (2.11–2.52 mmol/l) after unilateral thyroidectomy within 48 h or during the follow-up period and/or the need for vitamin D and calcium supplements. Complications lasting within and beyond 6 months were defined as transient and persistent, respectively. Contralateral lobe recurrence-free survival (RFS), loco-regional RFS, disease-specific survival (DSS), and overall survival (OS) were calculated in months.

### Statistical Analyses

Statistical analyses were conducted using SPSS, v.25.0 (IBM Corp., Armonk, NY, USA). Categorical variables were analyzed by the χ^2^ test and are presented as numbers with percentages. Continuous variables were analyzed by the Student’s t-test and are presented as the mean with standard deviation, or median and range, when appropriate. The Kaplan–Meier method was used to analyze the survival outcomes and calculate the 5-year survival rates. *p* < 0.05 was considered statistically significant.

## Results

### Comparisons of Patients and Tumor Characteristics

Of the 409 patients initially included, 39 patients with a follow-up of less than 12 months were excluded. Finally, 370 patients diagnosed with unilateral PTC after undergoing lobectomy and ipsilateral central-compartment neck dissection met the inclusion criteria for this retrospective study. Among them, 128 patients (128/370, 34.6%) belonged to the CN group. In the cohort, the median follow-up time was 62 months, ranging from 16 to 85 months. Excluding two patients who died from other disease at 16 and 18 months after initial surgery, the shortest follow-up time was 46 months. The median follow-up time for the UN and CN groups was 64 months (ranging from 16 to 85) and 57 months (ranging from 18 to 83), respectively (*p* = 0.609). The median age at diagnosis for all patients was 38 (ranging from 18 to 78) years old, and 314 (84.9%) patients were younger than 55 years old. Most patients were female (76.5%). Mean tumor size was 1.40 ± 0.96 cm and 154 patients (41.6%) had a tumor ≤ 1 cm. Microscopic ETE was observed in 23 patients (6.2%). Histological patterns of PTC included classical (82.7%) and follicular (17.3%) variants. All patients from both groups were classified as AJCC Stage I. There were no significant differences in gender (*p =* 0.189), PTC subtype (*p* = 0.232), or presence of microscopic ETE (*p* = 0.665) between the two groups. Patients in the CN group were older (*p* = 0.001) and had a larger tumor (*p* < 0.001) than those in the UN group. The proportion of patients older than 55 years old in the CN group was higher than that in the UN group (*p =* 0.02). Patients in the CN group had a higher frequency of tumors > 1 cm (*p =* 0.023). **Table 1** summarizes the patient and tumor characteristics.

**Table 1:** Comparison of patient and tumor characteristics between the UN and CN nodule groups.

	Total (n = 370)	UN group (n = 242)	CN group (n = 128)	*p*-value
Age				
Mean age	39.8 ± 12.8 y	38 ± 12.3 y	43 ± 13.1 y	0.001
<55	314 (84.9%)	213 (88%)	101 (78.9%)	0.020
≥55	56 (15.1%)	29 (12%)	27 (21.1%)	
Gender				
Female	283 (76.5%)	180 (74.4%)	103 (80.5%)	0.189
Male	87 (23.5%)	62 (25.6%)	25 (19.5%)	
Tumor size				
Mean size	1.40 ± 0.96 cm	1.26 ± 0.84 cm	1.67 ± 1.12 cm	<0.001
≤1cm	154 (41.6%)	111 (45.9%)	43 (33.6%)	0.023
1-4cm	216 (58.4%)	131 (54.1%)	85 (66.4%)	
PTC subtype				
Classical variant	306 (82.7%)	196 (81%)	110 (85.9%)	0.232
Follicular variant	64 (17.3%)	46 (19%)	18 (14.1%)	
Microscopic ETE				
Yes	23 (6.2%)	16 (6.6%)	7 (5.5%)	0.665
No	337 (93.8%)	226 (93.4%)	121 (94.5%)	
Median follow-up time	62 (16, 85) m	64 (16, 85) m	57 (18, 83) m	0.609

UN, unilateral nodule; CN, contralateral nodule; PTC, papillary thyroid carcinoma; ETE, extrathyroidal extension.

### CNs

The mean size of the contralateral lobe nodules detected by US in the CN group was 0.52 ± 0.32 cm, and 7.8% (10/128) were >1 cm. Six patients underwent FNA biopsy of the contralateral lobe nodules, and the results were benign (Bethesda II). The proportions of very low-suspicion, low-suspicion, and intermediate-suspicion nodules were 11.7%, 20.3%, and 62.5% respectively (**Table 2**). No patient had a high-suspicion sonographic pattern. There were seven patients who could not be classified due to the lack of echo intensity description in the US report.

**Table 2:** ATA sonographic pattern of CNs.

ATA sonographic pattern	128 (%)
Very low-suspicion	15 (11.7%)
Low-suspicion	26 (20.3%)
Intermediate-suspicion	80 (62.5%)
High-suspicion	0 (0%)
Unknown	7 (5.5%)

### Postoperative Complications

Among all of the patients, 10.5% (39 patients) had transient voice impairment and 3.8% (14 patients) suffered from persistent voice impairment. The most common voice impairment symptom was hoarseness (12.2% transient and 0% persistent), and the most common persistent voice impairment symptom was low-pitch voice (2.2% persistent). In addition, some patients developed hypocalcemia (2.4% transient and 0% persistent) and dysphagia (1.6% transient and 0.8% persistent). There were no significant differences in the frequency of complications between the two groups (**Table 3**).

**Table 3:** Postoperative complications of patients in the two groups.

	Total (n = 370)	UN group (n = 242)	CN group (n = 128)	*p*-value
Voice impairment				0.337
No	317 (85.7%)	207 (85.8%)	110 (85.9%)	
Transient	39 (10.5%)	28 (11.6%)	11 (8.6%)	
Persistent	14 (3.8%)	7 (2.9%)	7 (5.5%)	
Dysphagia				0.199
No	361 (97.6%)	234 (96.7%)	127 (99.2%)	
Transient	6 (1.6%)	6 (2.5%)	0 (0%)	
Persistent	3 (0.8%)	2 (0.8%)	1 (0.8%)	
Hypocalcemia				0.936
No	361 (97.6%)	236 (97.5%)	125 (97.7%)	
Transient	9 (2.4%)	6 (2.5%)	3 (2.3%)	
Persistent	0 (0%)	0 (0%)	0 (0%)	

UN, unilateral nodule; CN, contralateral nodule.

### Comparison of Recurrence and Survival Outcomes

After a median follow-up period of 62 months, 4.1% (10/242) of the patients in the UN group and 5.5% (7/128) of the patients in the CN underwent reoperation (*p* = 0.559). All of them had tumor recurrence proved by pathological analysis. **Table 4** shows the characteristics of these patients. One patient in the CN group received a second selective neck dissection 20 months after the second surgery and 62 months after the first operation due to loco-regional recurrence. At the end of follow-up, there was no evidence of disease in the two groups.

**Table 4:** Cases of patients with recurrence.

No.	Group	Site of recurrence	Time to recurrence (m)	Management of recurrence	Final outcome
1	UN	Contralateral lobe	49	CT + CCND	NED
2	UN	Contralateral lobe	14	CT + CCND	NED
3	UN	Ipsilateral level VI CLN	10	SND	NED
4	UN	Contralateral lobe	20	CT + CCND	NED
5	UN	Ipsilateral level II-V CLN	71	SND	NED
6	UN	Contralateral lobe, Ipsilateral thyroid bed, bilateral level II-VI CLN	59	CT+SND	NED
7	UN	Contralateral lobe, contralateral level VI CLN	39	CT + CCND	NED
8	UN	Contralateral lobe, contralateral level VI CLN	38	CT + CCND	NED
9	UN	Ipsilateral level II, III CLN	13	SND	NED
10	UN	Contralateral lobe	68	CT + CCND	NED
11	CN	Contralateral lobe	6	CT + CCND	NED
12	CN	Ipsilateral level II-VI CLN	10	SND	NED
13	CN	Contralateral lobe	10	CT + CCND	NED
14	CN	Ipsilateral level II, III, VI CLN	50	CT+SND	NED
15	CN	Contralateral lobe	40	CT + CCND	NED
16	CN	Contralateral lobe	63	CT + CCND	NED
17*	CN	Contralateral lobe	42	CT + CCND	NED

UN, unilateral nodule; CN, contralateral nodule; CT, completion thyroidectomy; CCND, central-compartment neck dissection; SND, selective neck dissection; NED, no evidence of disease.

*Patient No.17 underwent another selective neck dissection 20 months after the second surgery, and there was no evidence of recurrence during the 17-month follow-up period after the third surgery.

**Figure 1:**
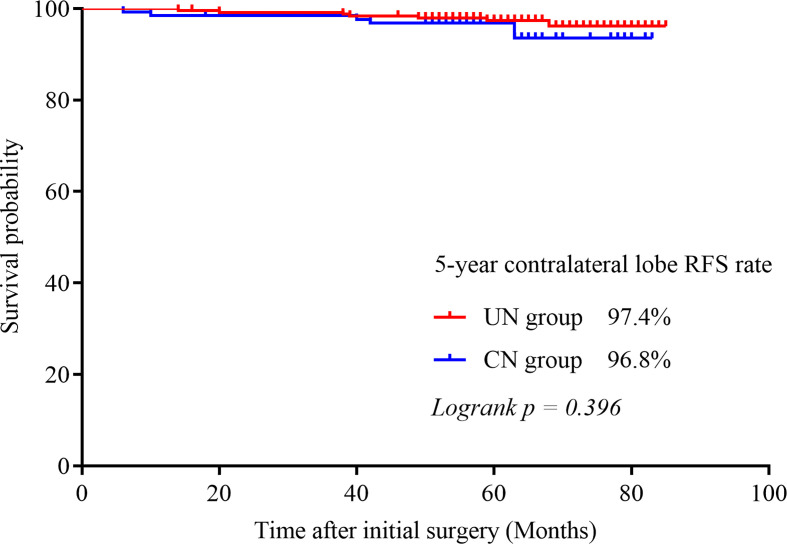
Kaplan–Meier survival curve illustrating the 5-year contralateral lobe RFS rate in both groups.

**Figure 2:**
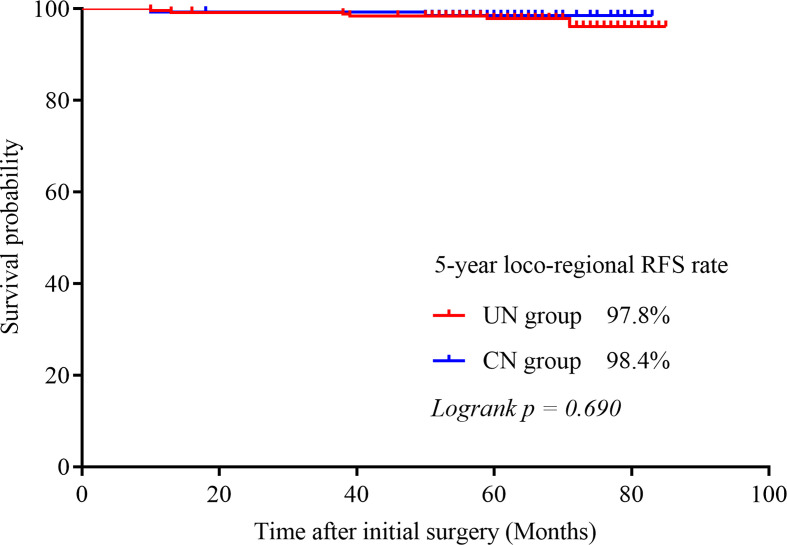
Kaplan–Meier survival curves illustrating the 5-year loco-regional RFS rate in the two groups.

Contralateral lobe recurrence was detected in 3.9% (5/128) of the patients in the CN group and in 2.9% (7/242) of the patients in the UN group (*p* = 0.601). All of them were proved to be PTC by pathologic analysis. The 5-year contralateral lobe RFS rate in the CN and the UN groups were 96.8% and 97.4% (*p* = 0.396), respectively (**Figure 1**). The rates of loco-regional recurrence were 1.6% (2/242) in the CN group and 2.5% (6/128) in the UN group (*p* = 0.564). The 5-year loco-regional RFS rates in the CN and UN groups were 98.4% and. 97.8%, respectively (*p =* 0.690; **Figure 2**). There were four non-disease-specific deaths: two patients in the UN group died from an unknown cause and breast cancer, respectively, and two patients in the CN group died from lung cancer and ovarian cancer, respectively. The 5-year DSS rates were both 100%, and the 5-year OS rates were 99.2% in the CN group and 98.4% in the UN group (*p* = 0.517).

## Discussion

Despite the sharp increasing incidence in PTC, the mortality rate has remained unchanged and the 20-year survival rate of these patients is close to 95% (20). It is widely recognized that the increasing incidence is largely due to overdiagnosis caused by the detection of subclinical cancers that would never cause harm (21), which certainly leads to overtreatment (22). Therefore, clinical guidelines are calling for low-intensity treatment of PTC (11, 12). Previously, total thyroidectomy was the main treatment for PTC, while unilateral thyroid lobectomy was only performed for patients with microcarcinoma (tumor < 1 cm) (23). However, in the past decade, studies have confirmed that patients with low- or intermediate-risk PTC who undergo total thyroidectomy have similar recurrence and survival rates as those who undergo unilateral thyroid lobectomy (6–9, 24). At present, most clinical guidelines consider unilateral thyroid lobectomy a valid treatment option for patients with tumor < 4 cm, non-gross ETE, and non-metastatic disease (10–12).

One of the benefits supporting this conservative trend is that patients after unilateral thyroid lobectomy have a lower incidence of postoperative adverse events. In a retrospective study of 62,722 patients, Hauch et al. (25) found that the overall rate of complications after unilateral thyroid lobectomy was 10.8%, which was significantly lower than that after total thyroidectomy (20.4%, *P <* 0.0001). The authors also found that the rate of complications after total thyroidectomy decreased with the surgeon’s experience, but even for high-volume surgeons (those performing > 99 thyroidectomies/year), this rate (14.5%) was still higher than that after unilateral thyroid lobectomy (7.6%, *p* < 0.0001) (25).

Moreover, many studies have shown that thyroid lobectomy is more effective than total thyroidectomy in reducing various postoperative complications such as hypocalcemia and recurrent laryngeal nerve injury (26–28). The incidence of persistent complications after unilateral lobectomy in our study was also much lower than total thyroidectomy reported in previous articles. The rate of persistent voice impairment, hypocalcemia, and dysphagia after unilateral thyroid lobectomy in our study were 3.8%, 0%, and 0.8%, respectively. While in different studies, the incidence of these three persistent complications after total thyroidectomy can be up to 31% (29, 30), 6.8% (31–33), and 20% (34, 35), respectively. More importantly, thyroid lobectomy does not cause the rare but extremely serious postoperative complication of bilateral recurrent laryngeal nerve paralysis, which generally results in severe dyspnea requiring tracheostomy (36).

Furthermore, patients undergoing total thyroidectomy must receive lifelong thyroid hormone replacement therapy, while thyroid lobectomy gives a chance to avoid this tedious daily task and its potential adverse effects (37). In addition to replacing endogenous thyroid hormone, the other purpose of hormone replacement therapy after thyroidectomy is to indirectly prevent recurrence or progression of thyroid cancer through negative feedback on pituitary thyroid-stimulating hormone (TSH) secretion (38). However, due to the lack of evidence that low-risk patients after lobectomy can benefit from postoperative TSH suppression, there is no consensus on postoperative hormone therapy for these patients (11). The 2015 ATA guidelines recommend that low-risk patients who undergo unilateral lobectomy maintain TSH level in the low to moderate normal range (0.5–2 mU/L), and patients with TSH in this range do not need thyroid hormone therapy (11). Kim et al. (39) showed that for low-risk PTC patients undergoing unilateral lobectomy, 34% did not need hormone replacement therapy and the remaining 66% used levothyroxine to maintain normal (rather than suppressed) TSH levels (0.86–4.69 mcIU/mL); after 5-year follow-up, recurrence was only detected in 1% of the patients. Similarly, Lee et al. (40) found that even for those who received TSH suppression therapy, 53.2% successfully stopped using levothyroxine when they maintained euthyroid status and only 0.3% of the patients had recurrence.

As discussed above, clinical guidelines are calling for more conservative extent of thyroidectomy for PTC, which raises the question of whether contralateral lobe nodules should still be an indication of total or completion thyroidectomy. Evidence supporting total or complete thyroidectomy is the probable high risk of contralateral malignancy in patients with unilateral PTC. Ibrahim et al. (16) studied 97 patients who were diagnosed with PTC after initial lobectomy and subsequently received completion thyroidectomy; the results showed that 48% (47/97) had contralateral lobe PTC. However, Ibrahim et al. (16) did not take into account the US features of the residual contralateral lobe. Wu et al. (17) analyzed the pathology of 347 cases of papillary thyroid microcarcinoma with ultrasonically benign-like CNs after total thyroidectomy, and found that 28.9% (100/347) had contralateral PTC. Notably, patients in these two studies underwent total or completion thyroidectomy, while those who received lobectomy alone were not considered; this selection bias could lead to a higher probability of finding a contralateral PTC. Recently, Ritter et al. (15) studied 112 PTC patients with solid contralateral benign or nonsuspicious thyroid nodules who underwent thyroid lobectomy alone; they found that after a median follow-up of 6 years, 11% (12/112) patients received completion thyroidectomy, and only 5% (6/112) were pathologically proven to have a PTC in the contralateral lobe.

In our study, we compared the outcomes between PTC patients with or without contralateral lobe nodules after unilateral thyroid lobectomy. Firstly, after a median follow-up period of 62 months, for patients with benign or nonsuspicious CN, the rate of clinical contralateral lobe PTC was only 3.9% (5/128) and the rate of loco-reginal recurrence was 1.6% (2/242). Second, in our study, there was no significant difference between patients with CN and patients without CN in terms of contralateral recurrence and loco-regional recurrence rate (5-year contralateral lobe RFS rates were 96.8% and 97.4%, respectively, *p* = 0.396; 5-year loco-regional RFS rates were 98.4% and 97.8%, *p* = 0.690); and the DSS and OS rates were similar between the two groups (5-year OS rates were 98.4% and 99.2% respectively, *p* = 0.517; 5-year DSS rate was both 100%).

In addition, a recent study by Sun et al. (41) showed that patients without nodules in the contralateral thyroid lobe at the time of initial lobectomy had a high probability, up to 41.9%, of developing nodules in the contralateral lobe, but only 6.5% of these nodules were proven to be clinically malignant. The rest of ultrasonically nonsuspicious new nodules achieved favorable clinical outcomes under ultrasound surveillance (41).

Consistent with the study from Ritter et al. (15) and Sun et al. (41), we proved that low-risk PTC patients with contralateral benign or nonsuspicious nodules have a low risk of developing clinical contralateral PTC. Our results further suggest that benign or nonsuspicious nodules in the contralateral lobe do not increase the risk of recurrence and disease-specific death. Moreover, completion thyroidectomy and (or) selective neck dissection remain timely and effective for recurrence found during follow-up. Therefore, though several studies have reported that PTC patients with CN have a high probability of contralateral PTC (16, 17), this favorable result suggests that if the contralateral lobe is removed without evidence of malignancy, it may lead to the overdiagnosis of contralateral subclinical PTC, which will certainly result in overtreatment to some extent.

Additional support for the aggressive surgical treatment is that the 2015 ATA guidelines recommend that PTC patients with contralateral thyroid nodules undergo total or completion thyroidectomy in order to carry out the postoperative radioiodine (RAI) therapy plan (11). However, several recent studies demonstrated that RAI therapy should not be routinely administered to patients with low-risk PTC. Ibrahimpasic et al. (42) reported that, after a follow-up time of 62 months, low-risk PTC patients managed without RAI had a similar RFS to those managed with RAI (96% *vs.* 100%, *p* = 0.337) after total thyroidectomy. According to a large population study of 276,558 patients, Orosco et al. (43) found that low-risk patients benefited the least from RAI treatment; by contrast, iodine therapy might even increase disease-specific mortality in low-risk T1a patients.

Although our study confirmed the benefits and safety of unilateral thyroid lobectomy for low-risk PTC patients with benign and nonsuspicious nodules, there is no doubt that postoperative lifelong follow-up is a challenge for both patients and physicians. In particular, some patients prefer total thyroidectomy as the initial treatment in order to avoid completion thyroidectomy for recurrence in contralateral thyroid lobe. Surgeons should take the patient’s preference and compliance of the postoperative follow-up into account when considering the extent of the thyroidectomy.

This study had some limitations. First, it was a retrospective and single-center study. Second, 39 patients were excluded due to insufficient postoperative follow-up data, which may have led to selection bias. Third, since the 30-year postoperative recurrence rate of differentiated thyroid carcinoma is up to 30% and 66% of recurrences occur in the first 10 years after operation (44), the follow-up period of our study may not be long enough to reveal the differences in recurrence rates between the two groups.

## Conclusion

PTC patients with benign or nonsuspicious CNs have similar recurrence and survival rates after unilateral lobectomy compared to those without CNs. Contralateral thyroid nodules alone should not be an indication for total or completion thyroidectomy.

## Data Availability Statement

The original contributions presented in the study are included in the article/supplementary material. Further inquiries can be directed to the corresponding author.

## Ethics Statement

Written informed consent was obtained from the individual(s) for the publication of any potentially identifiable images or data included in this article.

## Author Contributions

JL, JZ, SL, and HW conceived and designed the study. TM and HW conducted data collection and analyzed the data. TM wrote the manuscript. JL and TM revised the manuscript. All authors contributed to the article and approved the submitted version.

## Funding

This research was supported by the special fund for deep-underground medical research (Grant No YB2018002) and 1.3.5 project for disciplines of excellence (Grant No.ZYJC18016 and ZYJC21048) provided by West China Hospital, Sichuan University, as well as the Sichuan International Technological Innovation Cooperation Project (Grant No.2018HH0159), National Natural Science Foundation of China (Grant No.51822403 and 81800892) and the research fund of Health commission of Sichuan province (Grant No.20PJ029).

## Conflict of Interest

The authors declare that the research was conducted in the absence of any commercial or financial relationships that could be construed as a potential conflict of interest.

## Publisher’s Note

All claims expressed in this article are solely those of the authors and do not necessarily represent those of their affiliated organizations, or those of the publisher, the editors and the reviewers. Any product that may be evaluated in this article, or claim that may be made by its manufacturer, is not guaranteed or endorsed by the publisher.
